# Esthetically driven immediate provisionalization in the anterior zone: 5-year results from a prospective study evaluating 3.0-mm-diameter tapered implants

**DOI:** 10.1007/s00784-024-05832-x

**Published:** 2024-07-31

**Authors:** Paul Weigl, Georgia Trimpou, Pablo Hess, Martin Kolinski, Gionata Bellucci, Davide Trisciuoglio, Bertil Friberg, Sonia Leziy, Bilal Al-Nawas, Wilfried Wagner, Alessandro Pozzi, Liliana Ottria, Jörg Wiltfang, Eleonore Behrens, Christoph Vasak, Werner Zechner

**Affiliations:** 1 J. W. Goethe University, Frankfurt am Main, Germany; 2 Midwest Dental Implantology, St Charles, IL USA; 3 Maxillofacial Surgery and Dental Unit, Fondazione IRCCS Cà Granda Ospedale Maggiore Policlinico, Milan, Italy; 4 Brånemark Clinic Göteborg, Gothenburg, Sweden; 5 Pacific Perio Prosthetic Group, Nanaimo, BC Canada; 6 University Medical Center Mainz, Mainz, Germany; 7 University Tor Vergata, Rome, Italy; 8 University Clinic Kiel, Kiel, Germany; 9 University Clinic of Dentistry, Medical University of Vienna, Vienna, Austria

**Keywords:** Esthetics, Tapered-body implant, Immediate loading, Alveolar ridge, Torque, Permanent dental restoration

## Abstract

**Objectives:**

Evaluate the 5-year safety and efficacy of a narrow-diameter (3.0 mm) implant that was immediately provisionalized with a single crown in the maxillary lateral incisor or mandibular central or lateral incisor area.

**Materials and methods:**

An open, prospective, single-cohort, multicenter study was conducted, in which narrow-diameter implants were placed in fresh, healed extraction, or congenitally missing sites. All patients were required to meet strict criteria for immediate loading. The primary endpoints were marginal bone levels (MBL) and MBL changes (MBLC) from implant placement to 5-year follow-up. Secondary endpoints included cumulative 5-year survival and success rates, soft tissue health, and esthetic parameters.

**Results:**

A total of 91 implants were placed in 77 patients. The mean MBL remained stable from the 1-year (− 0.79 ± 0.73 mm, *n* = 75) to 5-year (− 0.74 ± 0.87 mm, *n* = 65) follow-up. A marginal bone gain of 0.11 ± 0.83 mm was observed from the 1-year to 5-year follow-up. The cumulative 5-year survival rate was 96.5%, and the cumulative 5-year success rate was 93%. The clinical parameters, including the modified plaque index, modified sulcus bleeding index, Jemt’s papilla index, and pink esthetic score improved throughout the 5-year study.

**Conclusions:**

The study demonstrated that narrow-diameter implants represent a safe and predictable treatment option for subjects suitable for immediate loading and with limited bone volume or limited inter-dental space.

**Clinical relevance:**

Narrow-diameter implants with immediate provisionalization can be considered for use to restore missing or damaged teeth with predictable functional and esthetic outcomes. This trial was registered with ClinicalTrials.gov (NCT02184845).

## Introduction

Due to the significant impact of the anterior teeth on esthetics, patients who experience frontal tooth loss often demand immediate solutions and have high expectations. The provision of esthetically satisfactory outcomes positively impacts the daily life performance of patients [1] and should be a predominant consideration in restoration planning. However, limited inter-dental space in cases of missing mandibular incisors and maxillary lateral incisors creates a compounding challenge, which necessitates the use of narrow-diameter implants. To address esthetic requirements as well as patient expectations, the treatment with these implants is often combined with an immediate loading protocol. Numerous studies concluded that immediately loaded implants not only display survival comparable to those that underwent conventional loading protocols [2–4] but are instrumental in shaping papilla, which is critical for the optimal esthetic results in the anterior region [5, 6].

Another important consideration is the timing of implant placement. Within the first three months following tooth loss, bone resorption begins to occur, resulting in alveolar ridge defects that can reduce the width of the alveolar ridge by up to 50% [7–9]. By contrast, immediate placement of an implant-supported prosthesis might preserve osseous and soft tissue framework yielding better esthetic outcomes [10] or even slow or prevent bone loss, serving as an acceptable and predictable treatment option, particularly when supported by grafting to cover any gaps between the socket walls and the implants [11–13]. Nonetheless, immediate implant placement is commonly not feasible, for example in cases with congenitally missing teeth or in which tooth loss had occurred months or years before dental implant therapy was considered. Such sites often present with limited bone volume, making the use of regular-diameter implants difficult or impossible. In these instances, apart from posterior regions, narrow-diameter (3.0 mm) tapered implants may be also indicated as a valuable treatment option for single or multiple-unit restoration (lower incisors or upper lateral incisors).

In addition to the need for a narrow diameter due to space limitations and patients placing high importance on the esthetic outcome of implants replacing the anterior teeth, the rehabilitation of these sites with 2-piece implants is preferred over 1-piece implant systems, for esthetic flexibility. In particular, a root-shaped emergence profile within the soft tissue layer can be achieved with a two-component implant using a prosthetic platform positioned at bone level. Compared to the round cross-section of a tissue-level implant, the latter ensures a natural-looking crown shape at the transition to the soft tissue. Similarly, the potential of changing bucco-lingual or mesio-distal direction during placement enables clinicians to adjust the position if they are not esthetically satisfied [14].

To evaluate the safety and efficacy of a 2-piece, variable-thread, tapered 3.0-mm-diameter implant placed to restore single missing teeth in the maxillary lateral incisor area or mandibular central or lateral incisor area with immediate function, we conducted an open, prospective, single-cohort, multicenter study. Implants were placed in fresh extraction sites, healed extraction sites, and sites with congenitally missing teeth. All patients included in this study were required to meet strict criteria for immediate loading, including smoking status, good oral health, and sufficient bone quality and quantity. All implants were immediately provisionalized with single crowns. The primary endpoints were marginal bone level (MBL) and MBL changes (MBLC) from implant placement to 5-year follow-up. Secondary endpoints included cumulative 5-year survival and success rates, soft tissue health, and clinical esthetic parameters.

## Materials and methods

### Ethical considerations

The study was performed according to the Declaration of Helsinki. Each investigator received Ethics Committee approval for their study sites. Each subject gave written informed consent. This trial was registered with ClinicalTrials.gov (NCT02184845).

### Study Design

This open, prospective, multi-center, single-cohort, clinical investigation was designed to include 11 private clinic, hospital and university study sites in Austria, Canada, Germany, Italy, Sweden, and the US. Patients in need of one or more single-tooth implant-supported restorations in the maxillary lateral incisor or mandibular central or lateral incisor areas and who met all inclusion criteria and none of the exclusion criteria were consecutively included. All implants were placed between March 10, 2011, and March 18, 2015. Patients were assessed at baseline (implant placement), 6 months, 1 year, 2 years, 3 years, and 5 years later. At all follow-up appointments, patients received radiographic examinations, soft-tissue assessments, and implants were evaluated for survival. The last subject’s last visit took place on June 4, 2020. All adverse events were recorded in the study database.

### Inclusion criteria

Patients were included if they provided informed consent; were at least 18 years old; required one or more single-tooth implants-supported restorations in the maxillary lateral incisor or mandibular central or lateral incisor areas; presented with natural tooth roots on both sides adjacent to the implant position; were physically and mentally capable of a study participation throughout the 5-year follow-up period; had sufficient bone volume at the implant site to support a 3.0-mm implant with a length of at least 10 mm; met the criteria for immediate provisionalization within 24 h, including a minimum insertion torque of 35 Ncm; had an implant site free from tooth remnants; were healthy and practiced good oral hygiene, and presented with a favorable and stable occlusal relationship. In cases in which implants were placed in fresh extraction sites, the extraction socket was required to present with at least 3 intact walls, with a dehiscence defect of up to 3 mm permitted on the fourth wall, and proper and thorough debridement of the extraction site was performed.

### Exclusion criteria

Patients were excluded if they were unable or unwilling to provide written informed consent; were not sufficiently healthy to undergo surgical treatment; were at any risk of negative mental outcomes in response to treatment, based on the patient’s history; presented with any disorders, such as tumors, bone disease, or prior irradiation, at the implant site; presented with evidence of ongoing infections, endodontic, or periodontal problems in the teeth adjacent to the implant site; had any history of alcohol or drug abuse; were heavy smokers (> 10 cigarettes/day); had uncontrolled diabetes or were diagnosed with diabetes and had a history of neglecting doctor’s recommendations; had any other disease or required medications that might influence the involved tissues, such as intake of bisphosphonates, treatment with heparin, osteogenesis imperfecta, or osteoporosis; presented with severe bruxism or other destructive habits.

### Implant placement

Implants were placed in either healed sites, fresh extraction sites, or sites with congenitally missing teeth. All surgical decisions were left to the discretion of the treating clinician on a case-by-case basis, including the use of medications, anesthetics, flap or flapless approach, and bone or soft tissue grafting. All implants had a 3.0-mm-diameter, a variable-thread tapered geometry (NobelActive, Nobel Biocare AB, Goteborg, Sweden), a moderately rough anodized surface (TiUnite, Nobel Biocare AB) and were placed according to the manufacturer’s recommendations. All implants were subjected to immediate provisionalization if they met the stability inclusion criteria (insertion torque of at least 35 Ncm without further rotation) and the treating clinician deemed it to be a suitable treatment option.

During the procedure, bone quality was assessed and scored as follows: 1, the jaw is almost completely comprised of homogenous compact bone; 2, a core of dense trabecular bone is surrounded by a thick layer of compact bone; 3, a core of dense trabecular bone (with favorable strength) is surrounded a thin layer of cortical bone; or 4, a core of low-density trabecular bone is surrounded by a thin layer of cortical bone. Bone quantity was also assessed and scored as described previously [15]: A, most of the alveolar ridge present; B, moderate residual ridge resorption; C, advanced residual ridge resorption, with only basal bone remaining; D, some basal bone resorption; or E, extreme basal bone resorption.

When necessary, bone grafting was performed using autogenous bone, anorganic bovine bone matrix (Bio-Oss, Geistlich, Wolhusen, Switzerland), or allograft particulate (Symbios, Dentsply, Waltham, USA). When required, soft tissue grafting was performed using predominantly autogenous connective tissue. For immediate provisionalization, various abutments were used, at the clinician’s discretion. Temporary crowns (acrylic, ceramic, or other materials) were retained with cement or screws and adjusted to avoid all occlusal contacts during both static and dynamic movements, resulting in a non-functional occlusion. All implants had proximal contacts, but no implants were bound or splinted to adjacent teeth or crowns. Final abutment selection and timing of definitive prosthesis placement (DPP) were determined by the treating clinician. Final abutments were titanium, either straight or angled 15°, and crowns (acrylic, ceramic, or metal-ceramic materials) were retained with screws or cement. No protective occlusal wafers were prescribed following surgery, and patients were advised to maintain a soft diet for 6 weeks and restrict biting, chewing, and other functional use of the treatment area for approximately 8–10 weeks. Following implant placement, patients were subjected to internal and external reviews to verify satisfaction of the study criteria.

### Primary outcome measures

The primary outcome measures were the 5-year peri-implant MBL and MBLC based on periapical radiographic examinations. Radiographic examinations were performed at baseline (immediately following surgery) and at each follow-up visit with a standardized long-cone parallel technique using a custom-made bite block. Analysis was limited to images that included both the implant platform and visible threads. An independent radiologist (University of Gothenburg, Gothenburg, Sweden) measured bone heights as the distance between the implant platform and the most coronal bone level. The implant diameter was used to calibrate the distance to 0.1-mm accuracy. The MBL and MBLC evaluation was based on paired radiographs and the values were presented as the average of the mesial and distal measurements for each implant site when both were available or just one, if only mesial or distal was recorded. Negative numbers indicate levels below the reference point (top of the implant platform), whereas positive numbers indicate levels above the reference point.

### Secondary outcome measures

#### Implant cumulative survival and success rates

An implant that was removed or fractured beyond repair was deemed a failed implant. Implant success criteria were based on the criteria suggested by van Steenberghe [16]. Specifically, successful implants were defined as any implants that did not result in local or systemic allergic, toxic, or gross infectious reactions; were able to anchor a functional prosthesis; did not show signs of fracture or bending; did not present with mobility when individually tested by tapping or rocking with a hand-held instrument; and did not show signs of radiolucency on intraoral radiographs taken perpendicular to the implant–bone interface.

#### Soft tissue parameters and adverse events

Soft tissue parameters including plaque accumulation, bleeding on probing, papilla index, and pink esthetic score (PES) as well as complications (both device-related and non–device-related adverse events) were recorded.

The soft tissues adjacent to each implant were assessed at baseline and each follow-up visit. Plaque accumulation was assessed using a modified Plaque Index (mPI) [17], which scores the presence of plaque from 0 to 3, with 0 indicating no detectible plaque, 1 indicating plaque detected when running a probe across the marginal implant surface, 2 indicating plaque visible to the naked eye, and 3 indicating an abundance of soft matter. Bleeding was assessed using a modified Sulcus Bleeding Index (mBI) [17], in which a periodontal probe is passed along the gingival margin adjacent to the implant. Bleeding is scored from 0 to 3, with 0 indicating no bleeding, 1 indicating visible but isolated bleeding in spots, 2 indicating blood forming a confluent red line on the margin, and 3 indicating heavy or profuse bleeding. Jemt’s papilla index [18] was used to assess the contour of the soft tissue adjacent to the implant. The papilla is scored from 0 to 4, with 0 indicating no papilla present, 1 indicating less than half of the papilla height present and a convex soft tissue curvature is observed adjacent to the implant crown and the adjacent tooth, 2 indicating half or more of the papilla height present without extended to the contact point between the teeth, 3 indicating an optimal soft tissue contour, and 4 indicating a hyperplastic papilla that covers too much of the implant or adjacent tooth. Both the mesial and distal papillae were evaluated independently, and the worse value was recorded for each implant. To calculate PES, the mesial papilla, distal papilla, soft tissue level, soft tissue contour, alveolar process deficiency, soft tissue color, and soft tissue texture were assessed by an independent expert (Medical University in Vienna, Vienna, Austria), using the parameters defined by Furhauser and colleagues [19]. The mesial and distal papillae were scored as complete, incomplete, or absent. All other variables were compared with a reference tooth (the contralateral tooth, or in congenital cases where the contralateral tooth was absent, the adjacent central incisor was used as a reference, an approach deemed as pragmatic by Dr Furhauser, personal communication) and scored from 0 to 2, with 2 being the best. Individual scores were summed to obtain the total PES, which ranged from 0 to 14, with 14 being the best. The overall PES values were recorded at the patient level.

All adverse events throughout the study period, including biologic, technical, and prosthetic complications, were reported, and they were also categorized as either device-related or non–device-related.

### Statistical analysis

The study was powered to detect an MBLC of 0.5 mm ± 1.0 nm, requiring a sample size of at least 65 patients calculated based on the reference group from a study with narrow implants [20]. To compensate for an expected withdrawal rate of 20% over the 5-year period and to ensure that an equal number of patients were evaluated at each participating clinic, 84 subjects were initially targeted to be included in the study.

All data collected from implant placement to 5-year follow-up were used for statistical analysis. No missing data were imputed or included in the statistical evaluations. MBLC and soft tissue remodeling were assessed by Wilcoxon signed-rank tests, frequencies, and change over time. Implant success rate and cumulative survival rate were evaluated by Kaplan–Meier analyses. Continuous variables (MBL, MBLC, PES) are presented as the mean ± standard deviation (SD), whereas ordinal variables (Jemt’s papilla index, mPI, mBI, soft tissue level) are presented as the frequency and percentage. All statistical calculations were performed using SPSS software version 25.0 (SPSS Inc., Chicago, IL, USA).

This manuscript was written according to the Strengthening the Reporting of Observational Studies (STROBE) guidelines (von Elm et al., 2014).

## Results

### Patient characteristics

In total, the cohort comprised 77 patients with a mean age of 40.9 years ± 18.9 years at the time of implant placement where over 90% had bone quantity of category A or B. A considerable fraction (25.9%) were young subjects with congenitally missing maxillary lateral incisors. However, a quarter of all patients suffered from allergies and 13% from serious illnesses, where almost half (49.4%) of the subjects took previous and/or concomitant medication (1–20 per patient) with 137 drugs reported in total. The population predominantly consisted of 49 women (63.6%), who received 58 implants, and 28 men (36.4%) who received 33 implants.

The smoking habits of each patient, including the approximate number of cigarettes smoked each day and any changes in smoking habits were recorded throughout the study. Three-quarters of patients never smoked, and 88.3% were non-smokers at the study onset. A summary of previous and ongoing illnesses, allergies, parafunctional tendencies, and changes in general health status and medication use were also monitored throughout the study. These and other baseline characteristics of the study population are presented in Table 1.

**Table 1:** Baseline characteristics

		*n* (%)	*n*-assessed
**Patients**		**77 (100%)**	**77**
Sex	*Female*	49 (63.6%)	77
	*Male*	28 (36.4%)	
Age	Mean ± SD	40.9 ± 18.9	77
	Range	18–80	
Medical history	Periodontitis	7 (9.1%)	77
	Diabetes type I & II	2 (2.6%)	
	Ongoing serious illnesses*	10 (13%)	
	Allergies	20 (26%)	
Smoking habits	Never smoked	58 (75.3%)	77
	Past smoker	10 (13%)	
	Current smoker ≤ 10 cigarettes/day	9 (11.7%)	
Implants per subject	1	63 (81.8%)	77
	2	14 (18.2%)	
**Initial Site Assessment / Soft Tissue Grafting / Implants & Surgical Approach**			**91** ^§^
Bone quantity	A	42 (46.2%)	91^§^
	B	41 (45.1%)	
	C	7 (7.7%)	
	D	1 (1.1%)	
	E	0 (0%)	
Bone quality	1	3 (3.3%)	91^§^
	2	31 (34.1%)	
	3	51 (56%)	
	4	6 (6.6%)	
Soft tissue grafting	Buccal and lingual flap	1 (1.1%)	91^§^
	Connective tissue graft	12 (13.2%)	
	Roll flap	1 (1.1%)	
	Tunnel technique	1 (1.1%)	
	None	76 (83.5%)	
Flap design	Flap with releasing incision	56 (61.5%)	91^§^
	Flap without releasing incision	15 (16.5%)	
	Flapless	20 (22%)	
Implant length (mm)	10	4 (4.4%)	91^§^
	11.5	16 (17.6%)	
	13	34 (37.4%)	
	15	37 (40.7%)	

^§^ *At implant level* * *Heart conditions, asthma, gastritis, Morbus Crohn, hip replacement*

### Implant parameters

The majority of implants (59, 64.8%) were placed in a lateral incisor site of the maxilla. The remaining implants (32, 35.2%) were placed in a central or lateral mandibular incisor site. Most patients (63, 81.8%) received one implant, with only 14 patients (18.2%) receiving 2 implants, each due to congenitally missing teeth, replacing both lateral maxillary incisors (FDI/EU positions 12 and 22). All implants had the 3.0-mm-diameter, and most were 13 mm (34, 37.4%) or 15 mm long (37, 40.7%). Of 91 implants, 67 were placed into healed or congenitally missing sites (73.6%). The 24 fresh extraction sites were monitored for infection at the time of implantation, with 5 (5.5%) sites displaying chronic infection and no sites presenting with acute infection. A flap approach was used for 71 (78%) surgeries, with or without a releasing incision. Prior to implantation surgery (≥ 3 months), bone grafting was administered at only 7 sites (7.7%) utilizing either autogenous bone (*n* = 2, 2.2%), allograft (*n* = 4, 4.4%), or xenograft (*n* = 1, 1.1%). Whereas during implant placement surgery, bone grafting was conducted for 20 (22%) implant sites, using predominantly xenograft (*n* = 18), allograft (*n* = 1), or autogenous bone (1 combined with xenograft, and 1 alone). During surgery, soft-tissue grafting was performed at 15 (16.5%) implant sites using mainly autogenous tissue. A minimum insertion torque of 35 Ncm was required for study inclusion, and 87 of 91 implants (95.6%) were inserted with the recommended final torque of 35 to 45 Ncm, with an average insertion torque across all implants of 39.03 ± 4.65 Ncm.

Provisional prostheses included immediate temporary abutments (36.3%), engaging temporary abutments (28.6%), esthetic abutments (12.1%) angulated esthetic abutments (13.2%), and others. Veneering for most of the temporary crowns was acrylic (95.6%), with just 4 implants receiving ceramic.

Definitive prosthetic restorations were placed in 74 patients (at 86 sites). Various abutments and retention methods were used for definitive prosthesis placement, as shown in (Table 2). Angulated screw channel (ASC) abutments were not available until toward the end of the study; therefore, most cases were cement-retained due to the importance of esthetics for anterior teeth, with only 11 abutments employing screw-based retention.

**Table 2:** Abutment vs. retention type at DPP

Retention	Cement	Screw
Abutment		
Procera Esthetic	7	1
Immediate Temporary	0	1
Narrow Profile	3	0
Esthetic	35	1
15˚ Esthetic	19	0
Other	11	8
**Total**	**75**	**11**

**Fig. 1:**
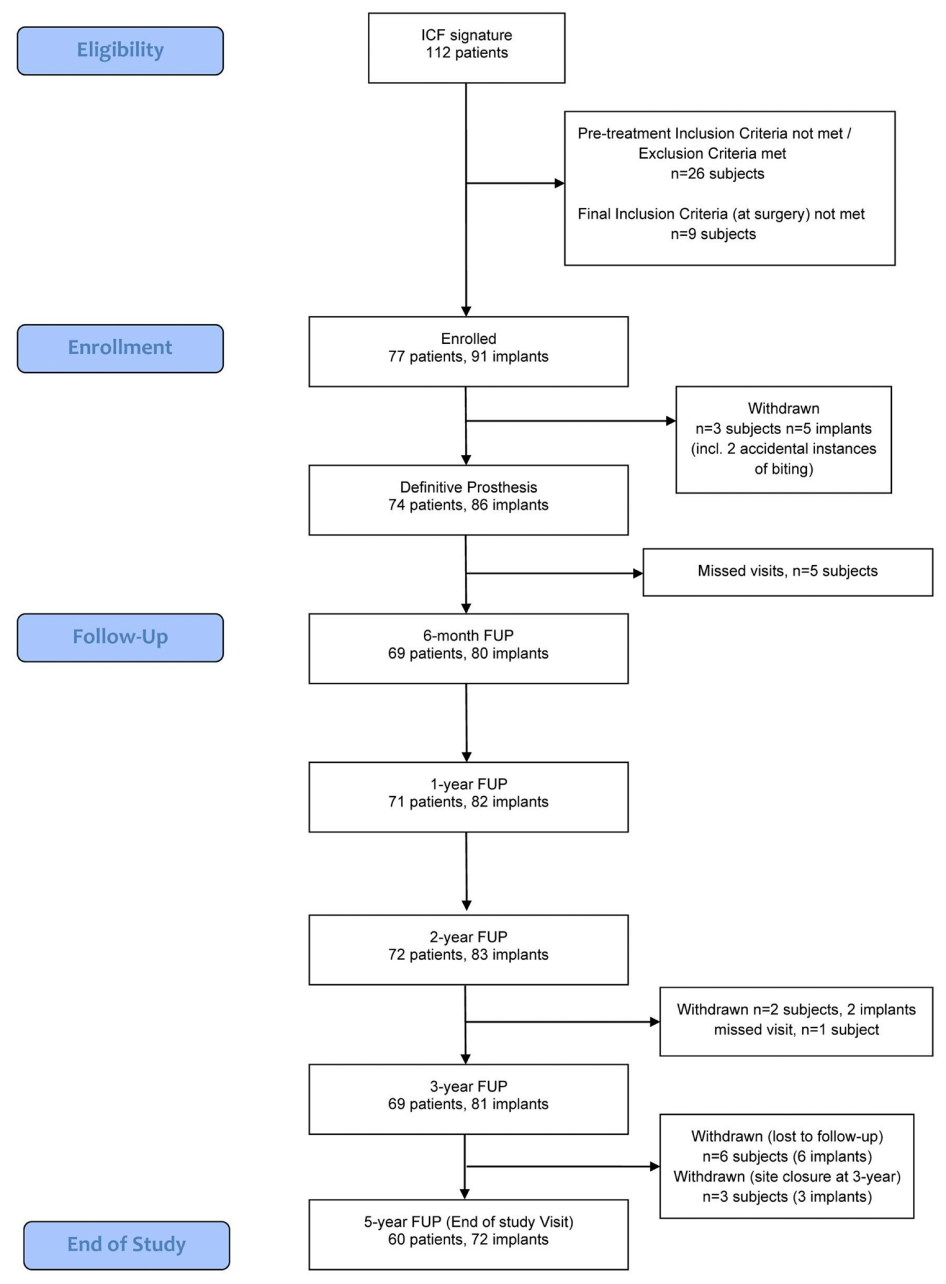
Treatment flow diagram (STROBE). Several patients with single or double implants missed different visits, of those, two subjects had their definitive prosthesis delivered at a 6-month follow-up, in these cases, only the DPP visit was counted

Between the implant placement and the 5-year follow-up visit, 17 patients (fitted with 19 implants) had withdrawn or were lost to follow-up, leaving a total of 60 patients and 72 implants in the final analysis. The flow diagram showing patient enrollment and withdrawals is shown in Fig. 1.

### Outcome measures

#### Primary endpoints: MBL and MBLC

**Fig. 2:**
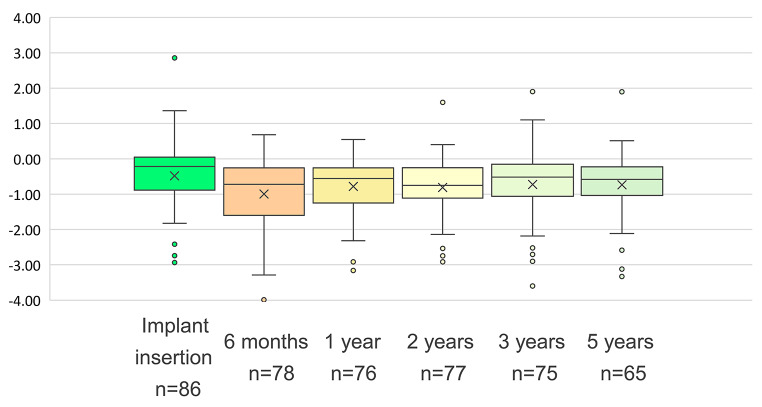
Marginal bone level assessed throughout the study. Box-and-whisker plot with means indicated as crosses and outliers as circles

The primary endpoint entailed evaluation of the marginal bone level changes from implant insertion to five years, based on paired radiographs. The mean 5-year MBLC in the current study was − 0.21 mm. At baseline, the mean MBL was − 0.48 ± 1.10 mm (*n* = 86). In the three cases in which implants were placed into fresh extraction sites, X-rays showed MBLs of − 6.0 mm at baseline, and these values were excluded from the analysis to avoid any false-positive bone gain calculated due to the extraction site morphology. At 6-month follow-up, the mean MBL decreased to − 0.99 ± 0.98 mm (*n* = 78). By 1-year follow-up, the mean MBL increased to − 0.78 ± 0.73 mm (*n* = 76). The mean MBL remained relatively stable from the 1-year to 5-year follow-up, with a 3-year mean MBL of − 0.72 ± 0.91 mm (*n* = 75) and a 5-year mean MBL of − 0.74 ± 0.87 mm (*n* = 65, Fig. 2).

From baseline to 6-month follow-up, an initial and statistically significant negative MBLC of − 0.50 ± 1.29 mm was observed. Subsequent MBLC from surgery to 1-, 3- and 5-year visits were respectively as follows: −0.24 ± 1.30 mm (*n* = 73), − 0.12 ± 1.40 mm (*n* = 69), and − 0.21 ± 1.29 mm (*n* = 63). Marginal bone gain of 0.11 ± 0.83 mm was observed from the 1-year to 5-year follow-up, with a minimal marginal bone loss of − 0.04 ± 0.55 mm from the 3-year to 5-year follow-up. No MBLC values after the 6-month follow-up were statistically significant.

#### Cumulative survival rate

Surviving implants were defined as any implants remaining in the jaw that were stable and offered anchorage to a functional prosthesis, whereas a failed implant was defined as any implant that required removal, was damaged beyond restoration, or otherwise failed to be classified as surviving. Throughout the 5-year follow-up period, a total of 3 implants placed in 3 subjects failed. One implant was removed 29 days post-surgery due to pain, and two implants were mobile and were lost on days 43 and 81. The cumulative 5-year survival rate was 96.5%.

#### Cumulative success rate

This study defined a successful implant based on a modified version of the criteria suggested by van Steenberghe [16]. A total of four cases were reported as unsuccessful throughout the 5-year study period, including the three failed implants described above, and a fourth implant that was mobile at the 5-year follow-up visit. The cumulative 5-year success rate was 93.0%.

#### Plaque index

Plaque accumulation at the marginal implant/abutment surface was assessed using the mPI, which revealed general improvements in oral hygiene throughout the study. Across the 5-year study, the majority of sites were scored as 0, indicating no detectible plaque: 65% at 6 months, 74.4% at 1 year, 71.6% at 3 years, and 73.6% at 5 years. Only 2 cases (2.8%) were scored as 3, indicating an abundance of soft matter, at 5 years. No significant differences in mPI scores were observed when comparing between time points across the 5-year study.

#### Bleeding index

Bleeding was assessed using the mBI. Across the 5-year study, most implant sites were scored as 0, indicating no bleeding: 75% at 6 months, 82.9% at 1 year, 79% at 3 years, and 73.6% at 5 years. Only 3 sites had worsening bleeding scores over the 5-year period, but no changes were statistically significant.

#### Jemt’s papilla score

**Fig. 3:**
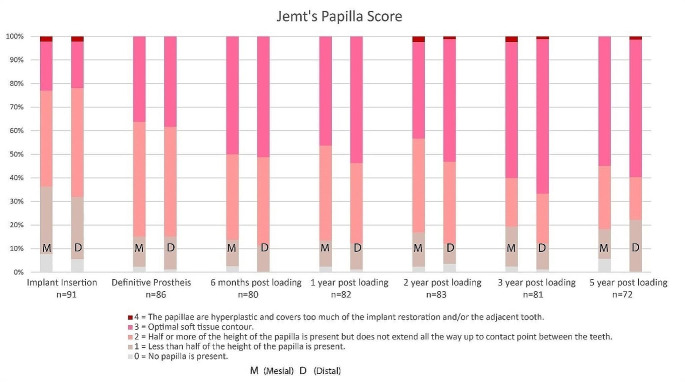
Jemt’s papilla index

From baseline to 5-year follow-up, the proportion of implant sites scored as 1, indicating less than half of the optimal papilla height was present, decreased from 28.6 to 11.0% for mesial papilla and from 26.4 to 11.0% for distal papilla. The proportion of implant sites receiving a score of 3, indicating optimal papilla presentation, increased significantly from 20.9 to 54.2% for mesial papilla and from 19.8 to 58.3% for distal papilla. Only four cases presented with mesial papilla scores of 0 at 5-year follow-up, despite good overall oral hygiene, with no signs of plaque formation or bleeding. Figure 3 shows the distribution of mesial and distal papilla scores across the study period.

#### Pink esthetic score

**Fig. 4:**
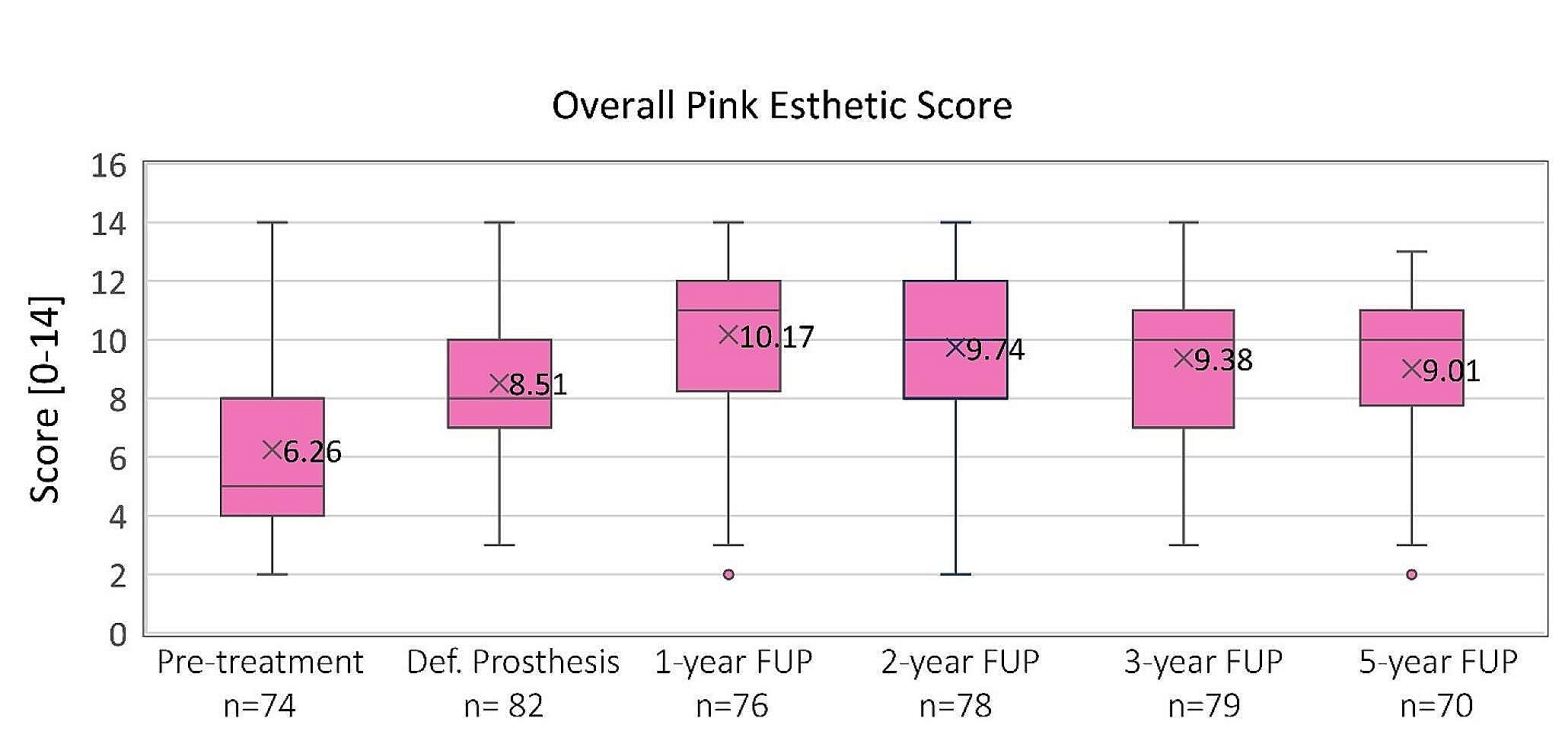
Pink esthetic score

Mean overall PES increased significantly from 6.26 prior to implantation to 9.01 at the 5-year follow-up. The soft tissue contour score showed the most improvement, increasing from 0.47 prior to implantation to 1.11 at the 5-year follow-up. The soft tissue level also showed good improvement, increasing from 1.00 prior to implantation to 1.53 at the 5-year follow-up. Figure 4 shows the mean overall PES scores across the study period.

#### Complications

Throughout the 5-year study period, 22 complications were recorded (Table 3). Of those, 6 were biologic, and 16 technical. Two patients with biologic complications, one with a single and one with two affected implant positions, required surgical intervention. The implantation sites showed signs of infection and inflammation and were accompanied by exudate, requiring laser-assisted peri-implantitis protocol (LAPIP) or flap surgery enabling debridement of the implant surface and the removal of granulation tissue. However, no marginal bone loss greater than 3 mm occurred in any of these cases, all three resolved, and the implants were considered successful at subsequent follow-up visits. In addition to the complications, 6 adverse events were recorded, none of them deemed as related to implants or study procedures. These included hospitalization (for arthroscopy intervention, Miller Fischer Syndrome, nasal bone fracture, knee surgery, and accident followed by immobilization with a support corset), and 1 patient was diagnosed with psoriasis.

**Table 3:** Biologic and technical complications throughout the study

Type	Description	Resolution
Biologic (6)	Serious infections/inflammation (2)	LAPIP/debridement surgery
	Post-op pain without swelling (1)	Not resolved with antibiotics; stop procedure
	Sulcular exudation (1)	Cleaning with ecoxeridine O2; crown polishing
	6mm-deep pocket (1)	Referral for periodontal treatment
	Non-osseointegration (1)	Failure; stop procedure
Technical (16)	Seating of impression coping screws (1)	Exchange of components
	Abutment screw fracture (3)	Replaced
	Screw fracture with crown mobility (1)	Replaced
	Accidental biting (2)	Implant explantation/spontaneous loss
	Implant mobility (1)	New device
	Crown fractures (2)	Replacement
	Crown and abutment fracture (1)	Replacement
	Accidental ceramic veneer fracture (fall) (1)	Provisional repair, subsequent replacement
	Central screw issue (1)	Replacement
	Crown loosening (2)	Healing abutment/re-cementation
	Abutment issue (1)	Replacement and new provisional crown

#### Outcomes and complications with cement- vs. screw-retained restorations

Overall, clinical outcomes with cement-retained restorations vs. crew-retained restorations were comparable, except for marginal bone loss in the initial remodeling phase which was more notable in implants with screw-retained restorations. Conversely, there was an absence of complications in this group. The details of outcomes per retention mode are provided in Table 4.

**Table 4:** Radiographic and clinical outcomes per retention type

	Cement-retained (*n* = 75)	Screw-retained (*n* = 11)
Primary outcome measure		
MBLC (mean ± SD)		
IP-6 months	-0.46 ± 1.32 (n = 67)	-0.75 ± 1.08 (n = 10)
IP-1 year	-0.16 ± 1.35 (n = 62)	-0.72 ± 0.79 (n = 11)
IP-3 years	-0.07 ± 1.49 (N = 59)	-0.45 ± 0.70 (n = 10)
IP-5 years	-0.19 ± 1.36 (N = 53)	-0.29 ± 0.83 (n = 10)
Secondary outcome measures at 5 years		
Implant survival (all sites/failed)*	75/0	11/0
Implant success (all sites/failed)	75/0	11/1
Plaque index (0/1/2/3)	49/8/4/1^#^	4/5/0/1^#^
Bleeding index (0/1/2/3)	48/9/5/0^#^	5/4/1/0^#^
Jemt’s papilla score-Mesial (0/1/2/3/4)	4/7/14/36/1^#^	0/2/5/3/0^#^
Jemt’s papilla score-Distal (0/1/2/3/4)	0/11/13/37/1^#^	0/5/0/5/0^#^
Overall PES (mean ± SD)	9.02 ± 2.33 (n = 60)	9.00 ± 2.45 (n = 10)
Complications		
Biologic (all sites/complications)	75/3^&^	11/0^&^
Technical (all sites/complications)	75/10^&^	11/0^&^

* *All 3 implant failures occurred before DPP* ^***#***^ *Number of sites with the corresponding score*

^***&***^*Of all complications*,* 3 biologic and 6 technical occurred before DPP*

**Fig. 5:**
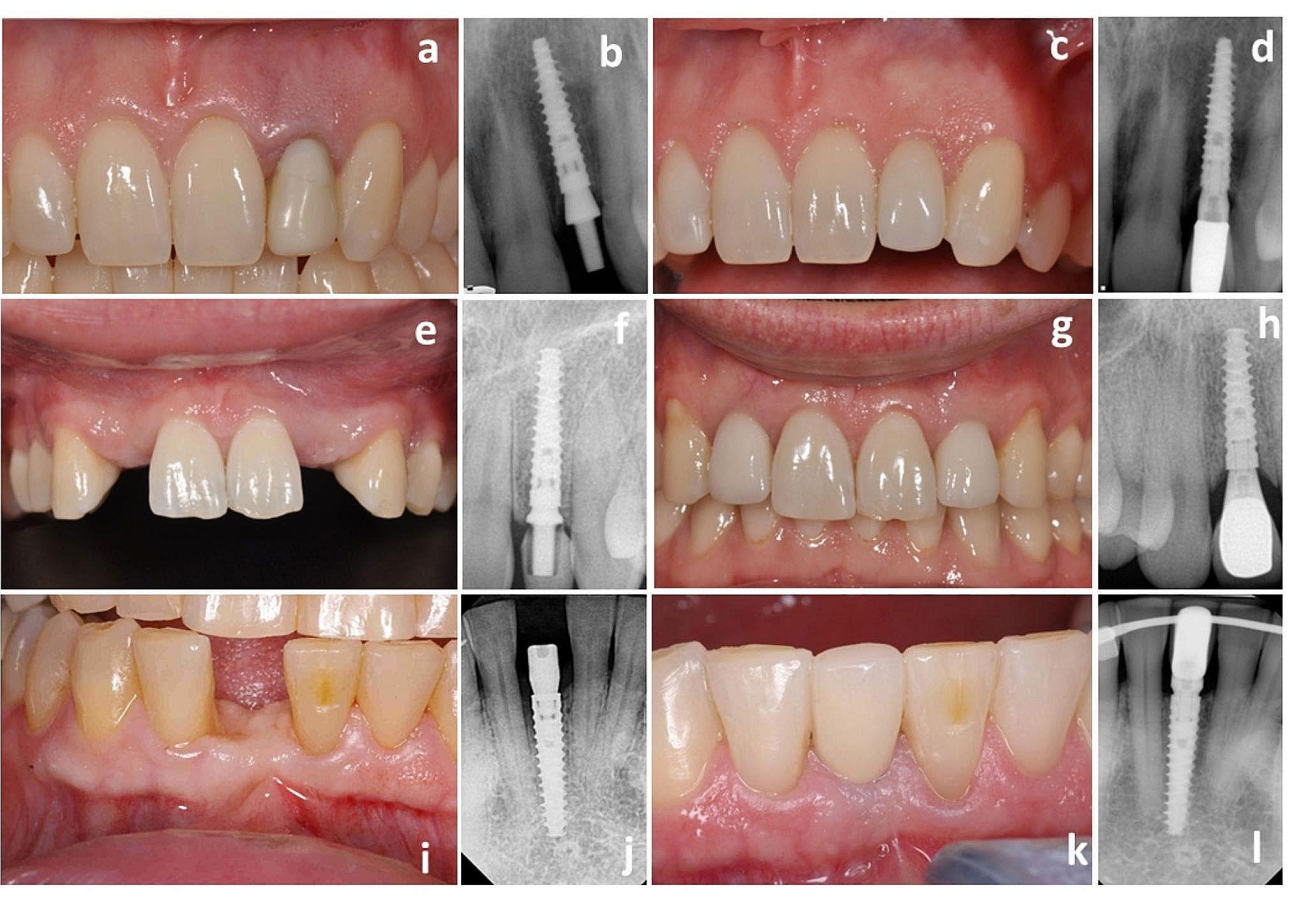
Clinical picture and radiographs of patients before treatment, at implant insertion, and 5 years later. Column on the left, images from pretreatment **(a, e, i)**, center left, radiographs taken at the insertion of narrow (3-mm-diameter) implant **(b, f, j)**, center right, photos at the 5-year follow-up visit after implant placement **(c, g, k)**, and on the right, corresponding radiographs **(d, h, l)**. Top row, a 38-year-old male **(a, b, c, d)** with a hopeless dentition at the lateral incisor in the maxilla (FDI position 22) received a 15-mm-long implant in the fresh socket. Middle row, a 27-year-old male **(e, f, g, h)** missing lateral incisors in the maxilla (FDI positions 12 and 22) received 13/15-mm mm long implants. Bottom, a 61-year-old female with a missing central incisor in a healed site of the mandible **(i, j, k, l)** received a 15-mm long implant

The 5-year follow-up visit occurred at 5.06 ± 0.22 years (range 4.75-6.00 years). Several clinical examples before treatment, at implant insertion, and 5-year follow-up are presented in Fig. 5.

## Discussion

This open, prospective, multi-center, single-cohort, clinical investigation evaluated the 5-year efficacy and safety of narrow-diameter (3.0 mm) implants using an immediate loading protocol for restoration in areas limited by interdental space or alveolar bone ridge width. The primary endpoint, which was the marginal bone level change over time (− 0.21 ± 1.29 mm between insertion and 5-year follow-up) revealed excellent bone stability and represents significant improvement in comparison to the reference study. This result is also better compared to the average of − 0.82 mm from 6 studies with narrow-diameter implants (a minimum follow-up of 36 months) calculated in a recent meta-analysis [21]. A previous study using a narrow-diameter (3 mm), but parallel-wall design implant reported a very comparable MBL change of − 0.11 ± 0.96 mm from surgery to 3 years and − 0.15 ± 0.95 mm to 5 years [22], suggesting that the compressive stress induced by the tapered, apical variable-thread, narrow-diameter implant used in this study did not pose an increased risk of bone loss. Furthermore, published research suggests that tapered and threaded design facilitates a better distribution of load and decreases buccal/facial incidence of perforation compared to cylindrical implants due to great surface area and more anatomical shape [23–25].

The 5-year cumulative survival rate of 96.5% reported in the current study was similar to the 97.3 ± 5% survival rate reported by a systematic review for implants with diameters of 3.0–3.25 mm [26]. In the present study, all 3 implant failures occurred within the first 3 months after implant insertion, with one implant removed due to pain at the insertion site and two implants removed due to mobility caused by accidental biting. The factor most commonly coinciding with implant failure is incomplete or inadequate osseointegration, as in the present study, where all failures occurred during the initial remodeling phase. This observation suggests that once the implants are able to achieve a stable implant–bone interface (or secondary stability), the risk of failure is much lower [27]. In order to minimize the risk of failures, particularly with immediately provisionalized restorations, primary implant stability could be enhanced through implant shape. Studies showed that taper-design implants display a higher rotational stability compared to cylindrically-shaped implants, especially in fresh-socket placement [28, 29]. In fact, a tapered narrow implant could be more suitable for sites with spongy trabecular bone, because it enables adequate primary stability in such soft, low-density bone in contrast to parallel wall-shaped implants [29, 30]. Furthermore, it is likely that, in the current study, trabecular bone transitioned into better quality, as supported by findings in pre-clinical investigations revealing rapid thickening of the cortical shell and the associated compensation for the observed loss of trabecular bone around implant after insertion [31–33]. On the molecular level, it may be related to an increased osteogenic response to mechanical loading [34].

No implant fractures were observed in the current study, despite the narrow 3.0-mm implant diameter and immediate provisionalization. This result is likely associated with an absence of high occlusal forces in the anterior zone, such as those characteristics of the molar region, especially with immediate provisionalization, [35, 36]. Consequently, these narrow-diameter implants are strongly counter-indicated in the premolar and molar regions where the bite force may be around three times greater than in the frontal area [37]. Despite concerns over the risk associated with immediate loading [38], implant survival rates are comparable to conventional loading [2–4].

The clinical parameters, including the mPI, mBI, Jemt’s papilla index, and PES improved or remained stable throughout the 5-year study, indicating healthy and viable long-term soft tissue response. For example, in contrast to the increase in bleeding on probing from 34.8 to 57.5% reported in the study with a similar patient population receiving 3.0-mm implants and with the same duration by Galindo-Moreno et al. [22], the number of sites with isolated bleeding spots was stable throughout the current study. The healthy soft tissues were reflected by the good esthetic outcomes. Since the narrow-diameter implants are used in the anterior zone where the alveolar bone ridge may be narrower or there is less interdental space, esthetics were of high importance to both clinicians and, especially to this relatively youthful cohort. Soft tissue preservation and pink esthetics were prioritized by clinicians, who generally planned to use the least invasive surgical approach possible, with minimal or no flaps [39]. Fresh extraction sites also tended to be prepared by removing teeth without flaps. The use of instant provisionalization has been shown to be essential for optimizing esthetic outcomes, particularly the height and appearance of the papilla [5, 6, 40].

An unexpected complication was screw fractures due to over-torquing. Most clinicians are accustomed to using 35 Ncm for implant placement, which is also a requirement for study inclusion; however, the screws themselves should not be tightened beyond 20 Ncm. The confusion between the torque values required for implant placement vs. abutment screw tightening brought about 5 fractures, revealing the importance of suitable training of the clinicians with respect to different steps of narrow-diameter implant placement.

The comparison of retention mode (cement vs. screw) revealed that most of the post-DPP clinical outcomes were comparable between the two groups with a couple of exceptions. One category pertained to the number of adverse events, where in contrast to cement-retained restorations, the screw-retained group had none. This agrees with the systematic review, which reported higher complication rates for cement-based retentions [41]. The other involved a more pronounced marginal bone loss surprisingly at the screw-retained restorations. This comparison needs to be interpreted with caution, however, since it was statistically underpowered to test the two modes of retention. Nevertheless, these results suggest that cementation can result in good clinical outcomes. A recent RCT similarly demonstrated comparable outcomes with the two retention modes, emphasizing the role of careful removal of excess cement [42].

This study has several limitations. Since it was conducted across multiple sites and involved different surgeons, it might have introduced some variability through the level of individual experience or the use of additional procedures, such as different techniques to create surgical flaps during implant placement, which could influence healing and long-term success. However, the goal was to assess the safety of effectiveness of this approach under real-world circumstances, and the overall outcomes were positive, indicating that this approach was not sensitive to specific expertise or techniques to impact the success. Furthermore, enrollment was not limited to a particular site type (healed, fresh extraction, or sites with congenitally missing teeth) and it allowed additional grafting procedures when required to produce the best outcome for the patient. The choice of grafting materials might have also impacted implant success and stability. The decision to use soft tissue-grafting procedures was made on a case-by-case basis at the treating clinician’s discretion. Similarly, the selection of grafting techniques could have influenced implant esthetics and stability, but the results were generally successful, regardless of any individual differences. Although the study was not powered to determine whether any of the above-mentioned factors have influenced the outcomes, it demonstrated the universality of the approach and its tolerance toward variations.

Due to otherwise strict inclusion criteria, nearly a quarter of approached patients were precluded from enrollment, and additional subjects who did not attain the minimum torque of 35 Ncm required for immediate loading protocols were excluded. Furthermore, the mean age of the study population was relatively low, with over a quarter of young patients aged between 18 and 28 years old who received implants in place of their congenitally missing lateral maxillary incisors. According to the American Academy of Implant Dentistry, the median age of implant wearer is 52 years old, more than a decade older than the average patient in the current study.

## Conclusion

This 5-year study confirmed that tapered-shape, variable-thread, 3.0-mm-diameter implants represented a safe and predictable treatment option for subjects with limited bone volume and/or limited inter-dental space, who qualified for immediate loading yielding healthy soft-tissue response and esthetically satisfactory results.

## Data Availability

The data that support the findings of this study are available from the study sponsor upon reasonable request.
